# Five material tissue decomposition by dual energy computed tomography

**DOI:** 10.1038/s41598-022-21193-5

**Published:** 2022-10-12

**Authors:** Maximilian E. Lochschmidt, Melina Gassenhuber, Isabelle Riederer, Johannes Hammel, Lorenz Birnbacher, Madleen Busse, Tobias Boeckh-Behrens, Benno Ikenberg, Silke Wunderlich, Friederike Liesche-Starnecker, Jürgen Schlegel, Marcus R. Makowski, Claus Zimmer, Franz Pfeiffer, Daniela Pfeiffer

**Affiliations:** 1 Chair of Biomedical Physics, Department of Physics, School of Natural Sciences, Technical University of Munich, 85748 Garching, Germany; 2 Department of Diagnostics and Interventional Radiology, School of Medicine, Klinikum Rechts der Isar, Technical University of Munich, 81675 Munich, Germany; 3 Munich Institute of Biomedical Engineering, Technical University of Munich, 85748 Garching, Germany; 4 Institute for Advanced Study, Technical University of Munich, 85748 Garching, Germany; 5 Department of Diagnostic and Interventional Neuroradiology, School of Medicine, Klinikum Rechts der Isar, Technical University of Munich, 81675 Munich, Germany; 6 Department of Neurology, School of Medicine, Klinikum Rechts der Isar, Technical University of Munich, 81675 Munich, Germany; 7 Department of Neuropathology, School of Medicine, Klinikum Rechts der Isar, Technical University of Munich, 81675 Munich, Germany

**Keywords:** X-rays, Tissues, Fluids, Computational methods, Imaging and sensing

## Abstract

The separation of mixtures of substances into their individual components plays an important role in many areas of science. In medical imaging, one method is the established analysis using dual-energy computed tomography. However, when analyzing mixtures consisting of more than three individual basis materials, a physical limit is reached that no longer allows this standard analysis. In addition, the X-ray attenuation coefficients of chemically complicated basis materials may not be known and also cannot be determined by other or previous analyses. To address these issues, we developed a novel theoretical approach and algorithm and tested it on samples prepared in the laboratory as well as on ex-vivo medical samples. This method allowed both five-material decomposition and determination or optimization of the X-ray attenuation coefficients of the sample base materials via optimizations of objective functions. After implementation, this new multimodal method was successfully tested on self-mixed samples consisting of the aqueous base solutions iomeprol, eosin Y disodiumsalt, sodium chloride, and pure water. As a first proof of concept of this technique for detailed material decomposition in medicine we analyzed exact percentage composition of ex vivo clots from patients with acute ischemic stroke, using histological analysis as a reference standard.

## Introduction

A clinically well-established method that enables material decomposition in general is dual-energy CT (DE-CT)^1^. There are several options for DE-CT, such as dual-source CT (DS-CT), rapid kVp-switching, and dual-layer CT (DL-CT), all of which are well-known options in the clinical setting^2–9^. For this study, a DL-CT scanner system was used, which differs from the other DE-CT options mainly in the type of detector arrangement. Here, the detector consists of two superimposed detector layers, resulting in a better detector response function for low-energy photons in the first layer and for high-energy photons in the second layer^10^. Thus, DL-CT provides detector-based energy separation with only one primary X-ray spectrum.

**Figure 1:**
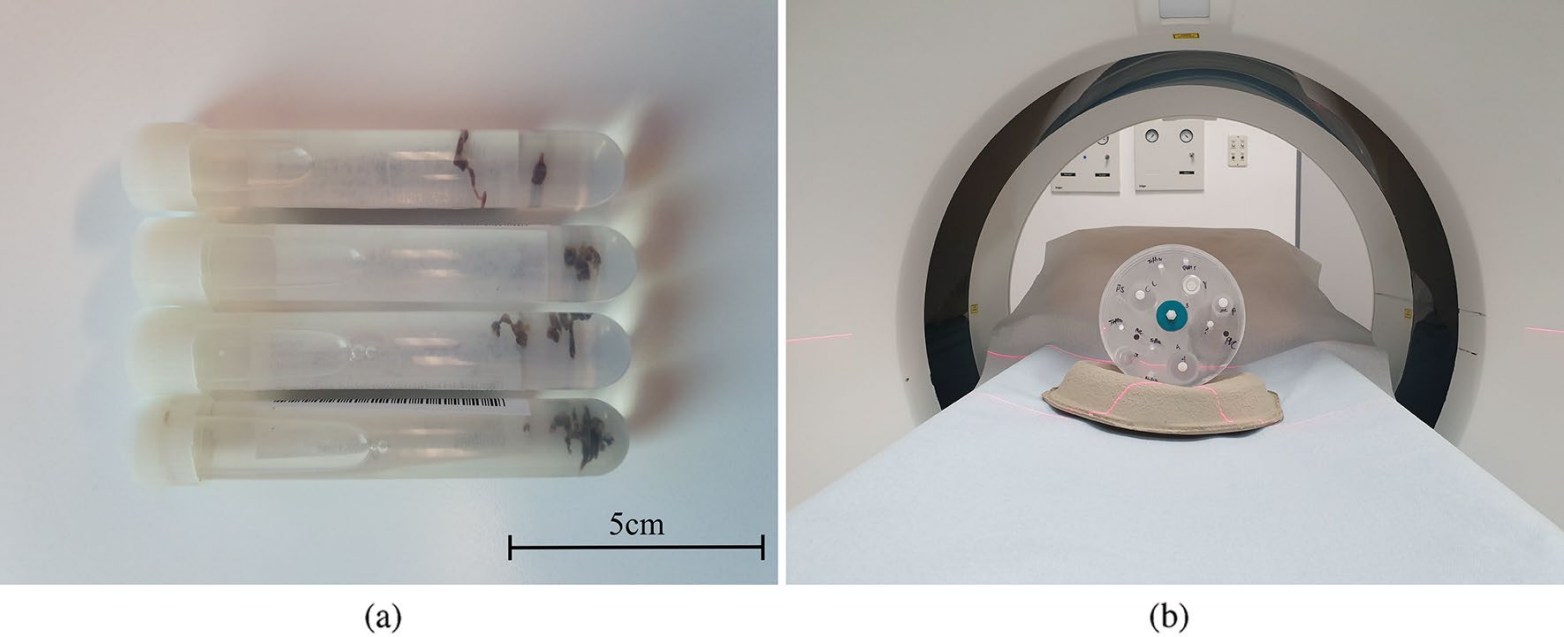
(**a**) Illustration of the basic measurement setup, showing clots stored in tubes filled with formalin after they were extracted from the patient by mechanical thrombectomy and immediately fixated with formalin. (**b**) For the measurement, two of these tubes can be inserted and measured by the dual-layer CT using a specimen holder. The specimen holder itself was placed on a cardboard tray to prevent the holder from moving during the measurement and to create a slightly inclined plane so that the clots remain at the bottom of the tubes.

In addition, due to the superimposed detector layers, both images are perfectly registered, so no post-processing registration is required. Using the measurement information gathered from the energy responsive detector, the detector response function and the X-ray source spectrum, virtual monoenergetic (monoE) images can then be calculated. Due to the fact that the total attenuation at one specific energy can be expressed as a sum of all individual mass attenuation coefficients multiplied with their densities, solving a linear equation system leads to material decomposition which has already been discussed in the scientific community regarding quantification, accuracy and methodology^11–14^. However, a mathematical limit is reached at a decomposition of two different materials by using two virtual monoE images and solving a simple linear equation system of two equations. A third equation at a further different energy would mean linear dependency and thus an infinite number of solutions of the system^15,16^.

Nevertheless, the determination of more than three materials is possible. For this, in addition to the virtual monoE information, volume conservation must be introduced as an additional condition and at the same time a more complex method is required. To this end, one concept has already been developed separating more than three materials. This method is using a triplet library. To create this library, several material triplets from a unlimited number of basis materials are created and used, with the help of which each voxel with the information of two virtual monoE attenuation coefficients is tried to be split into the respective three materials. Once this is done for each material triplet and voxel, only the correct physical results are then counted for the final solution^17^. However, this methodology is not suitable for specimens with high intermixing of all basis materials and at the same time contains low concentrations of basis materials. If two measured specimens are investigated, one of which consists only of basis materials A, B and C, and the other of which also contains a low concentration of basis material C on top, the attenuation values of both may still be in the same material triplet for which the material C is not taken into account. Thus, the amount of basis material C cannot be determined. Another generally occurring problem is that the X-ray attenuation coefficients of chemically complicated basis materials may not be known and,furthermore, cannot be determined by other or previous analyses. To solve these problems, we developed an algorithm that uses the information from two virtual monoE images at different energies as well as the data of a further modality, which can determine the percentage distribution of at least three basis materials. This new method then allows both five-material decomposition and can determine or optimize X-ray attenuation coefficients of the specimens’ basis materials via optimizations of objective functions. In addition, the algorithm is able to determine small concentrations of basis materials.

In particular, the newly developed algorithm was first verified with the aid of several test samples mixed in the laboratory and measured on the DE-CT, which consisted of four base materials of different but known concentrations. For this purpose, in addition to the knowledge of the exact composition of the samples, initial situations could be simulated in which components of the solution are not known. The algorithm managed to reliably determine the actual composition based on the simulated samples with incorrect knowledge about the composition and using virtual MonoE images. Based on this successful test of the method, it could now be applied to human ex-vivo clots consisting of red blood cells (RBC), white blood cells (WBC), the conglomerate of fibrin and platelets (fibrin/platelets)^18,19^, enriched iodine containing contrast agent^20^ and formalin which cannot be neglected in terms of X-ray attenuation here^21^. In this particular case, the histological findings provided the baseline information on RBC, WBC and fibrin/platelets. Whereas the information about enriched iomeprol and formalin is unknown. After the absorption properties of RBC, WBC, fibrin/platelets, iomeprol and formalin could be investigated and optimized by minimizing a cost function, the algorithm was able to determine the composition of all five basic materials for human clots. This application area is particularly interesting for medical diagnostics since knowledge of the exact composition of the clots originating from patients with acute ischemic stroke (AIS) can have a positive effect on the treatment and extraction of these^22^. It should be noted that this multimodal method can generally be used for soft tissue samples, biopsies and plaque as well.

**Figure 2:**
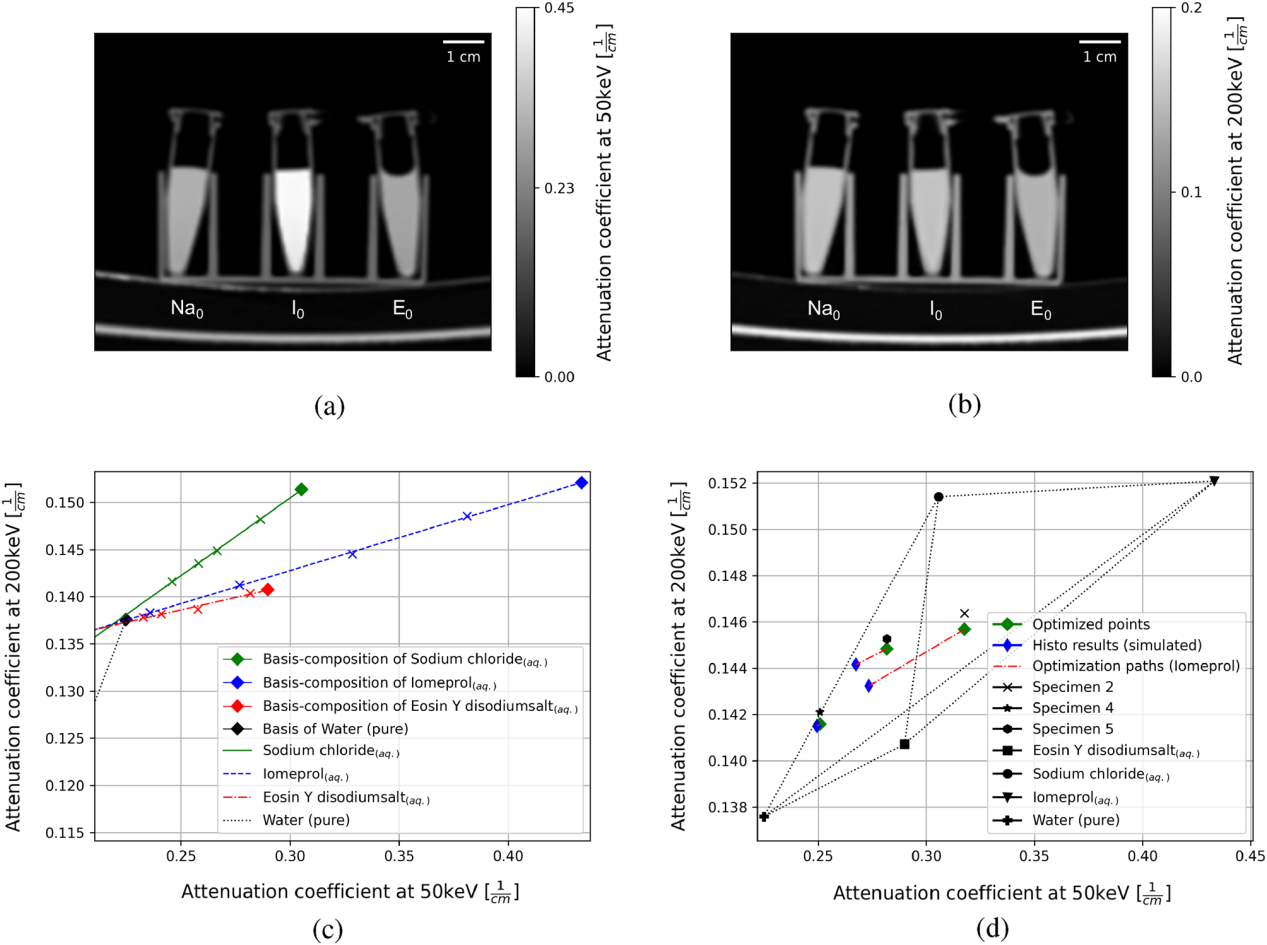
(**a**) Virtual monoE images at 50 keV and (**b**) 200 keV of the three basis compositions sodium chloride (Na$_0$), iomeprol (I$_0$) and eosin Y disodiumsalt (E$_0$). (**c**) The linear relationship of each basis material is shown for different concentrations mixed together in aqueous solution. Linear regressions were then created for the linear dependencies, allowing the endpoints of each basis material to be better determined computationally and with greater statistical accuracy. (**d**) The way of iodine optimization along a characteristic optimization path (red vector) is shown for specimen 2, 4 and 5. The exact results found by the algorithm are shown in Table 1.

## Results

### Model test for iomeprol correction

With the help of the DE-CT results of the concentration series of eosin Y disodiumsalt, sodium chloride and iomeprol, the ground truth attenuation coefficients could be determined from linear regressions. These resulted in $\mu _{Iomeprol}^{50} = 0.433\, 1/{\rm cm}$, $\mu _{Iomeprol}^{200} = 0.152\, 1/{\rm cm}$, $\mu _{Eosin}^{50} = 0.290\, 1/{\rm cm}$, $\mu _{Eosin}^{200} = 0.141\, 1/{\rm cm}$, $\mu _{NaCl}^{50} = 0.306\, 1/{\rm cm}$ and $\mu _{NaCl}^{200} = 0.151\, 1/{\rm cm}$ with higher statistical certainty compared to a single measurement of the basis maximum concentrations, which then would result in $\mu _{Iomeprol}^{50} = 0.434\, 1/{\rm cm}$, $\mu _{Iomeprol}^{200} = 0.152\, 1/{\rm cm}$, $\mu _{Eosin}^{50} = 0.290\, 1/{\rm cm}$, $\mu _{Eosin}^{200} = 0.141\, 1/{\rm cm}$, $\mu _{NaCl}^{50} = 0.305\, 1/{\rm cm}$ and $\mu _{NaCl}^{200} = 0.151\, 1/{\rm cm}$. The virtual monoE images at 50keV and 200keV of all three basis compositions are shown in Fig. 2a,b. The relative deviation from the mean value of the fit parameters were between 0.29% and 1.26% for 50 keV and except for the eosin y disodiumsalt which had a relative error of 8.14%, between 0.46% and 1.65% for 200 keV. The relative errors of the intercept of the linear regression was for all basis materials and energies between 0.10% and 0.18%. To visualize the property of linear dependency, the regressions and the considered single values of the attenuation at 200keV are plotted above the attenuation at 50keV for each measured concentration mixture and basis material Fig.2c. The results of the iomeprol fraction optimization for the testing specimens of aqueous mixtures with all basis materials are shown in Table 1. In addition, it is shown how the algorithm, based on the histological results, is shifted by an individual vector for each specimen towards the base material value of iomeprol until the difference between the shifted value and the CT value is minimal. This is shown for specimen 2, 4 and 5 (Fig.2d). It should be noted that the model was successfully tested on 19 samples for this research project, in addition to the 15 samples used to create the linear regression. Table 1 lists the most important results. The results of all measured samples can be found as Supplementary Table S6 and within the Supplementary Information as Figs. S2 and S3 online.

**Table 1:** Volume fractions to which the algorithm should converge as well as the fractions in parentheses for which the algorithm found the optimum.

Specimen	1	2	3	4	5	6
**Eosin Y**						
disodiumsalt$_{(aq.)}$	0.333 (0.330)	0.250 (0.242)	0.091 (0.090)	0.033 (0.034)	0.083 (0.083)	0.005 (0.005)
Iomeprol$_{(aq.)}$	0.000 (0.009)	0.250 (0.275)	0.000 (0.005)	0.033 (0.008)	0.083 (0.086)	0.003 (0.012)
Iomeprol$_{(aq.)}$ [$\frac{mg}{ml}$]	0.000 (0.180)	5.000 (5.400)	0.000 (0.100)	0.660 (0.160)	1.660 (1.720)	0.060 (0.240)
Water (pure)	0.333 (0.330)	0.250 (0.242)	0.909 (0.904)	0.667 (0.684)	0.417 (0.415)	0.496 (0.492)
Sodium chloride$_{(aq.)}$	0.333 (0.330)	0.250 (0.242)	0.000 (0.000)	0.267 (0.274)	0.417 (0.415)	0.496 (0.492)

The corresponding concentration is also given for the volume fraction of iomeprol and the optimization for samples 2, 4 and 5 is shown visually in Fig.2d.

### Iodine correction for human clots

#### Optimization for basis materials

**Figure 3:**
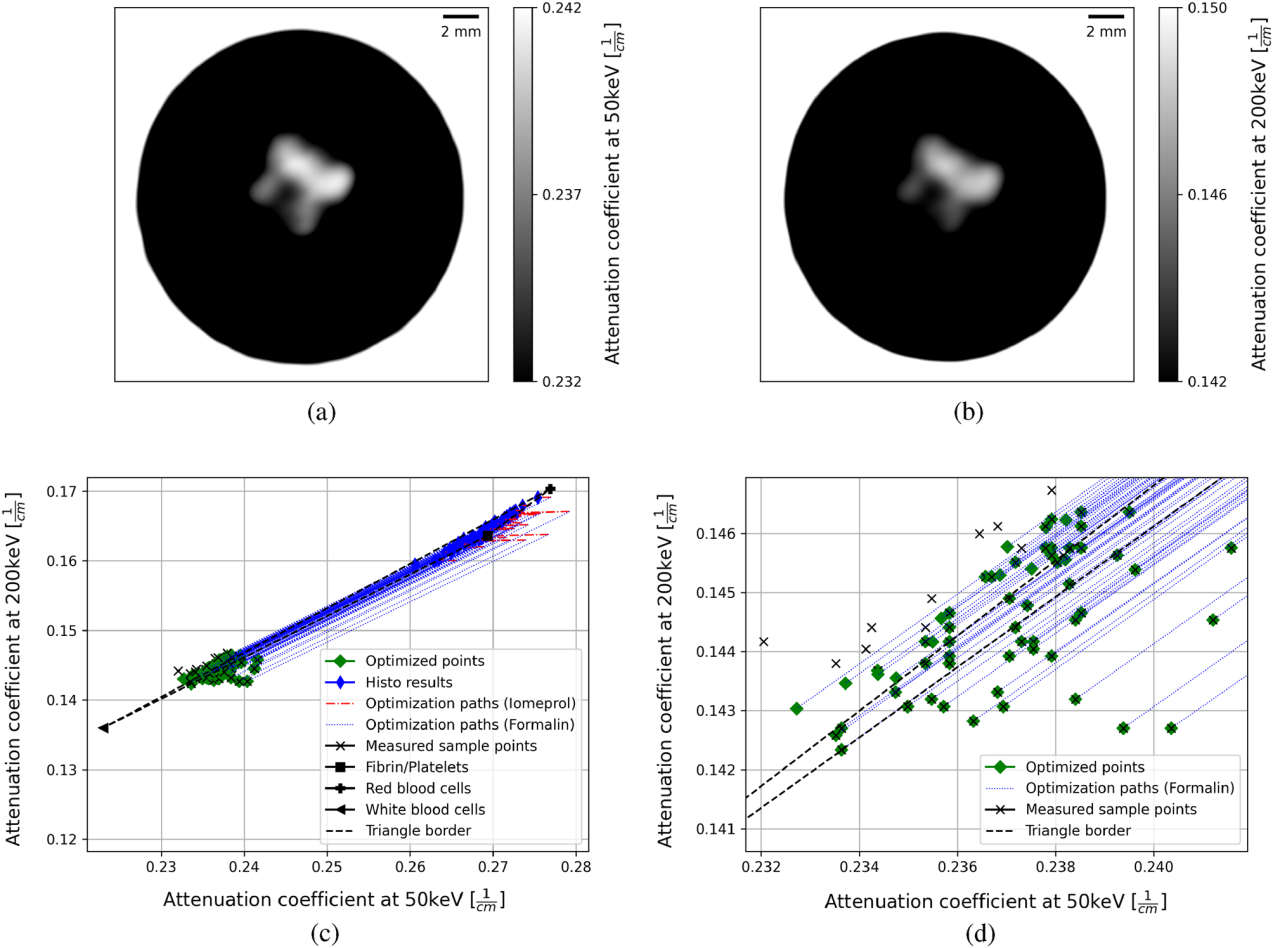
(**a**) Virtual monoE images of one measured clot at 50keV and (**b**) 200keV. (**c**) Illustration of the iomeprol as well as formalin correction using scatter plots and (**d**) magnification to the area visually representing the error between the optimized point and the value measured on the DE-CT. Using the basis material attenuation of fibrin/platelets, RBC, and WBC as well as the results of the histological analysis, a histological result is plotted in the scatter plot for each specimen. Starting from this point, the algorithm corrects the histological result point by the iomeprol and formalin concentrations along the vectors characteristic for each specimen (optimization paths). The algorithm then reaches its optimum as soon as the distance between the optimized point and the measured specimen point is minimal. The triangle boarder line indicates the enclosed area within which a specimen consisting exclusively of the three base materials can lie.

As an representation of all 73 measured clots, ones DE-CT result is shown for both virtual monoE images in Fig.3a,b. Before optimizing the attenuation coefficients of the basis materials, it has been important to determine a good starting value for each to make the optimization faster. This was successfully achieved with the help of the NIST database and PubChem^23,24^. The molecular formula and the densities of fibrin, iomeprol and formalin were looked up and by using the NIST database the attenuation coefficients at 50keV and 200keV were calculated. For RBC, the molecular formula could also be determined. But in this case by analyzing the exact composition of its protein structure. Again, the mass attenuation coefficients were determined using the NIST database using the atomic composition and the mean corpuscular hemoglobin concentration (MCHC) as density. For WBC, soft tissue was assumed and was taken directly from the NIST database. The exact results of these initial values can be found online in the Supplementary Information as Fig. S4, Tables S3, S4 and S5. Based on these initial values, the algorithm found the optima of the correction factors at cost function values of 0.9‰ for sample group one, 1.4‰ for sample group two, and 0.8‰ for sample group three. The correction factors and final weighted attenuation coefficients are listed in Table2.

**Table 2:** Correction factors of the attenuation coefficients for the basis materials fibrin/platelets, RBC and WBC.

Basis material name	Fibrin/Platelets	RBC	WBC	Fibrin/Platelets	RBC	WBC
Energy (keV)	50	50	50	200	200	200
Correction$_{Group1}$ (a.u.)	0.9128	3.7418	1	0.8657	3.7399	1
Correction$_{Group2}$ (a.u.)	0.9836	3.6263	1	0.9401	3.6118	1
Correction$_{Group3}$ (a.u.)	0.9180	3.9191	1	0.8736	3.9229	1
Weighted mean of correction (a.u.)	0.9311	3.7874	1	0.8859	3.7813	1
Weighted mean of attenuation [$\, 1/{\rm cm}$]	0.2693	0.2769	0.2230	0.1636	0.1703	0.1361

This is listed separately for each group consisting of four measured clots. The lower part of the table shows the final resulting attenuation properties considering the correction factors above. Furthermore, the mean value of the groups weighted by the inverse cost function value is listed. The values of the cost function were 0.9‰ for group one, 1.4‰ for group two and 0.8‰ for group three rounded to the fourth digit.

#### Iodine correction

**Figure 4:**
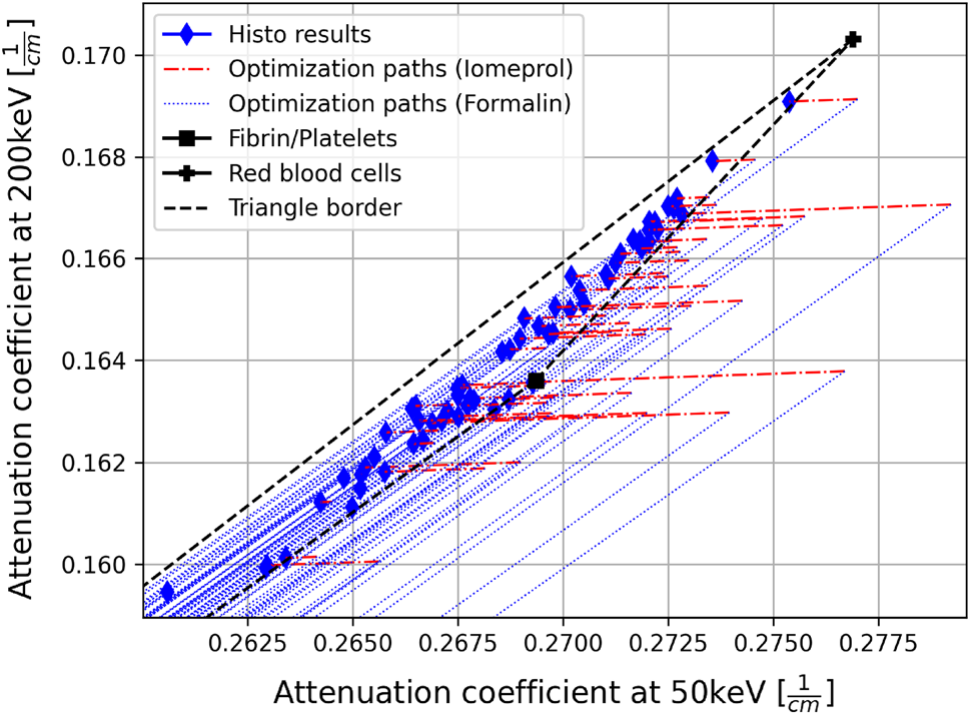
Illustration of the section from Fig.3c showing the iompeprol correction calculated by the algorithm. The length of the optimization paths directly correlate to the iompeprol concentration.

Using the attenuation properties of RBC, WBC, and fibrin/platelets, the algorithm for the decomposition of five materials was applied to additional clots, resulting in the iomeprol concentration and the error between the optimized point and the measured point. These results are listed in Tables 3 and 4. Furthermore, the optimization and the matching between optimized and measured point is visualized in Fig. 3. To highlight the correction caused by the iomeprol content a section of the particularly interesting area is shown (Fig. 4). Results of all individual 61 clots can be found as Supplementary Table S7 online.

**Table 3:** Listing the mean of all iomeprol concentrations except the clots used for the optimization for the basis materials and the mean matching errors for these clots separated in the 50keV and 200keV part.

Mean iomeprol concentration (mg/ml)	Mean matching error (50keV) (%)	Mean matching error (200keV) (%)
0.32	0.075	0.127

**Table 4:** Representative four samples in which a sufficiently high iomeprol concentration has resulted.

Specimen	A	B	C	D
Iomeprol concentration (mg/ml)	1.231	1.172	1.061	0.899
Fibrin/platelets fraction (%)	99.16	66.12	48.76	34.38

In addition, the corresponding volume fractions of fibrin/platelets are given.

## Discussion

Five different materials have been separated and quantified from each individual clot in this study, based on the information of DE-CT combined with histological analysis. It should be mentioned that other imaging devices, such as a micro-CT, have much better resolution than a DE-CT used in the clinic. However, such devices are mainly used only on ex vivo specimens and have a much smaller field of view (FOV), which makes it impossible to scan patients as well. Although the samples on which the theory and applicability were tested are suitable in size for micro-CT, the aim of this research project is to demonstrate the in vivo applicability of this new method on the clinically established DE-CT. The in-house developed optimization algorithm showed a high accuracy for the iomeprol correction of the self-mixed specimens consisting of the water based basis materials namely iomeprol, eosin Y disodiumsalt, sodium chloride and pure water. The fact that the linear regression (Fig.2c) finds its optimum at low error of all fit parameters shows that the linearity of the systems of equations can be assumed. In addition, the fact that all linear regressions intersect well in one point and that this intersection point simultaneously corresponds with high accuracy to the theoretical comparison value of water shows that a systematic error can be excluded. This accuracy is very dependent of the iomeprol concentration. The specimen containing a iomeprol concentration of $1.66 \, {\rm mg/ml}$ was optimized to a concentration of $1.72 \, {\rm mg/ml}$ which means a relative error of $3.61\%$. By contrast, for a concentration of $0.660 \, {\rm mg/ml}$ the algorithm terminates at a value of $0.160 \, {\rm mg/ml}$ that means a relative error of $75.8\%$. These results substantiate the developed and implemented theory and show that the algorithm can result in high accuracy. In contrast, it shows that not every concentration is optimized with the same accuracy. In general this concentration dependency of the accuracy of the algorithm makes it difficult to decide which results are trustful and which are not. But now after testing the model with known concentrations and volume fractions of each specimen, it turned out that every concentration below $0.660 \, {\rm mg/ml}$ cannot be measured precisly. It is worth noting that this concentration boarder coincide with results of other previous studies that showed a boarder between 1.0 and 0.8 $\, {\rm mg/ml}$^25^. What has also been confirmed is the initial assumption that the triplet library method does not seem suitable for use on well-mixed samples with low iomeprol concentrations. It can be seen, especially for Specimen 5 in Fig.2d, that although there is iomeprol in the sample, the point is only shifted within the base-material triangle, which does not include iomeprol. The triplet library method would therefore not detect iomeprol even though it is present in the sample. The triplet-library method works well for mixtures of materials that have a cluster and are not strongly mixed. Now we know that clots in high resolution histological images show a cluster of the three main basic materials RBC, WBC and fibrin/platelets. Therefore, it can be assumed that the triplet library method should theoretically work. Due to the voxel size (0.41 mm × 0.41 mm × 0.8 mm) of this DE-CT measurement and the small size of the clots, the segmented clots appear intermixed. In addition, the typical iomeprol concentrations are very small. Thus, it is still reasonable that this method cannot work for clots. All this shows that the newly developed method of this study is of great importance for the field of material decomposition on clots.

The error of the specimens containing an iomeprol concentration of above $0.660\, {\rm mg/ml}$ can have a few origins. The sum of all typical errors occurring by mixing different concentrations can be seen in Fig.2. Since all concentrations of iomeprol, eosin Y disodiumsalt and sodium chloride are water based, the linear regressions should in theory cross the basis point of pure water exactly. What can be seen here is that the regressions crosses pure water slightly behind. Reasons can be the neglected volume excess that always occurs at mixing different media volumes^26^, error of the balance and volume change due to temperature^27^ change between the lab (293.15K) the specimens where mixed and the room temperature at the DE-CT setup. Since both the specimens used as basis material mixtures and the specimens with different basis substances and concentrations were prepared in the laboratory at the same time and were not separated from then on, the error should be small. It should be mentioned that due to the absorption of photons, characteristic radiation is also emitted by the samples themselves. However, this phenomenon is negligible here, since this form of secondary radiation occurs over the entire solid angle and most of the radiation that would hit the detector is absorbed by the anti-scatter grid in front of the detector. Moreover, considering the transition energies of all base materials of the measured clots and test mixtures, the energetic bandwidth reaches from below 1 keV to a maximum of 28 keV, which was calculated using the NIST database of transitions^28^. The highest transition energy is due to the iodine-containing contrast medium. Measurements showed that even the fluorescence of iodine samples in aqueous solution contributes only a small peak with a maximum of 20 counts on an energy bandwidth of about 27keV - 32keV and is very low compared to the Compton scattering of water^29^. Considering these points, it can therefore be assumed that the characteristic radiation of the samples does not significantly distort the measurement results. In summary, despite small and almost unavoidable sources of error, the accuracy of the theory and the implemented algorithm are of high quality and thus an application to human clots is justified and promising. Concerning the previously presented testing of the method, it is important to note that for the basis materials chosen for this purpose, whose K-edges are partly within the measured X-ray spectrum, theoretically further equations with spectral information at further monoenergetic energies would allow a material decomposition beyond the three materials by simply solving the linear system of equations. However, the outlook of this work is on the applicability to human blood clots, whose element with the highest nuclear charge number in vivo is iron with a nuclear charge number of 26. For the measured ex-vivo clots, the enriched iodine-containing contrast agent may then be added, but at the same time formalin is added as a fifth material due to the fixation process. Thus, these samples consist of four materials whose K-edges are not in the measured X-ray spectrum and one material for which this is true. However, this fact makes it impossible to obtain the material composition on the basis of further spectral information by simply solving a linear system of equations. Since the model is intended to test the applicability to human blood clots, the test mixtures were treated in the same way as biological samples whose K-edges do not lie in the relevant X-ray range.

Correction for iomeprol and formalin also worked well for the human clots with high accuracy. Also in this case, it is important to discuss the degree of linearity of the equation system based on the results. Although the individual base materials for human clots are difficult or even impossible to measure due to the difficulty of extracting the individual materials, the high degree of linearity can be indirectly observed from the optimization of the base material attenuation coefficients of fibrin/platelets, RBC and WBC. The small value of the cost function (Eq. 5) between 0.8 and 1.4% at the global optimum of several test samples shows that the approach of the cost function, in which the equation system was assumed to be linear, is justified. Thus, the model with the linearity approach could be applied to more samples regarding the iomeprol and formalin corrections. The mean relative matching error between the optimized point and the measured point is $0.075\%$ (50 keV) and $0.127\%$ (200 keV). For specimens that could be corrected by characteristic formalin as well as iomeprol vectors, it is not surprising that the error is exactly zero. This is due to the fact that in a 2D plane, each point or vector can be represented by two different vectors. Therefore, these two mean relative errors were calculated using specimens that could only be optimized by the characteristic vector of formalin and therefore did not necessarily result in an error of zero. The fact that the error is nevertheless small shows emphatically the high accuracy of the algorithm. The results also showed a mean iomeprol concentration of $0.320\, {\rm mg/ml}$, which is well below the limit of $0.660 \, {\rm mg/ml}$ set by the previously performed model test. Thus, for the vast majority of clots, an iomeprol concentration was determined that should be considered equal to zero, or at least lower than the detection limit. However, four clots were optimized to iomeprol concentrations between 0.899 ${\rm mg/ml}$ and 1.23 ${\rm mg/ml}$. These are above the limit of $0.660\, {\rm mg/ml}$ and thus are trustworthy results. From a clinical point of view, it is particularly interesting here that three of the four iodine-positive samples also had a comparatively high fibrin/platelets content. This correlation confirms previous studies that were also able to demonstrate this fact qualitatively^30–34^. Moreover, it can be seen that iomeprol does not accumulate to high levels in fibrin/platelets-containing clots randomly. Rather, a proportionality is discernible. For specimen A, which has a fibrin/platelets content of 99.2$\%$ as determined by histology, an iomeprol concentration of 1.23 ${\rm mg/ml}$ was determined. Up to specimen D, for which an iomeprol concentration of 0.899 ${\rm mg/ml}$ with a fibrin/platelets content of 34.4$\%$ was determined, the iomeprol concentration decreases directly proportional to the fibrin/platelets content (Table 4). It can be concluded that determination of the fibrin/platelets content in the clot may be possible by determination of the iomeprol concentration. It should be noted, that the clots were stored in formalin for different lengths of time from extraction to measurement. The storage times for specimens A, B, C and D, rounded up to the full hour, was 77 h, 56 h, 33 h and 31 h, respectively. Thus, it is possible that the iomeprol washed out of the clots differently. In this case, an exact protocol would have to be carried out with exactly the same and shortest possible storage times. When analyzing the results of all 61 clots, it is noticeable that there were significantly more clots that had similar or much higher fibrin/platelets content. Therefore, it is initially surprising that the results are not similar to those of samples A, B, C and D. One explanation for this could be that the components RBC, WBC and fibrin/platelets appear very regionally as clusters in the range of histological resolution. Therefore, the position of the clot in the artery may be decisive. If mainly a fibrin/platelets containing area of the clot is in contact with the artery wall, the iomeprol may diffuse in more difficulty or less. Since it is known from fluid dynamics that vortices can form behind an obstacle if this obstacle is held in a flow^35^, it may also play a role whether a fibrin/platelets-containing area is facing the blood stream or away from it. For the side of the clot facing the blood stream, a constant iomeprol concentration can be expected, since the blood is constantly circulating here. On the side facing away from the blood flow, flow-dynamic vortices can form, where a lower iomeprol concentration can be expected. Based on all the results of this ex vivo study, it can be concluded that an in vivo method might be possible if further studies will be done with in vivo clots. Such a methodology might be based on using two virtual monoE images for which no contrast agent has yet been administered to the patient. Thus, neither contrast medium nor formalin is present in the clot. This could then allow the separation into the respective volume fractions of RBC, WBC and fibrin/platelets with the aid of the three-material decomposition, for which volume conservation is considered.

Possible sources of error cannot be ruled out, which are mainly found in histological analysis, in the model simplification for the algorithm and in the segmentation of specimens. The histological analysis, which optically calculates the percentage of RBC, WBC and fibrin/platelets, has an accuracy of 89–$99\%$ output by the instrument. This internal error refers to how well the device was able to segment with the help of the training and not whether correct areas were necessarily segmented. Since such sections were excluded manually, the error is considered to be small. As the dimensioning of the cost function had to be reduced for the optimization of the basis materials, WBC was assumed to have a similar composition to soft tissue and was excluded from the optimization. Since the fraction of WBC in the clots is significantly lower compared to fibrin/platelets and RBC, the error is not weighted as heavily in comparison. However, it must be noted that for some clots a WBC fraction of 15$\%$ is quite possible. It can therefore not be excluded that the simplification of the model can play a role with regard to the total error of the analysis. Another source of error can be the segmentation process. Great care was taken to segment only the clot. But it cannot be completely ruled out that a few background voxels were also segmented. Nevertheless, the results show a very high accuracy. Thus, these errors are also small as claimed for the model testing. Another interesting way to verify the decomposition is to make a measurement on monoenergetic lines, whose energy would then have to correspond to the effective energy of the X-ray spectrum of the DE-CT. Since the X-ray tube of the DE-CT can only emit polychromatic spectra, external and non-clinical facilities such as a synchrotron would have to be used. In theory, this is a feasible method for further verification. However, this method is extremely difficult to perform, especially for clinical DE-CTs, since neither the input spectrum nor the detector response, nor the raw data of the detector layers are freely available.

## Methods

### Theory

In general, if mixtures consisting of three basic materials have to be decomposed, this can be achieved with knowledge of the attenuation properties of the individual materials by means of two virtual monoE images calculated on the basis of a DE-CT measurement and by considering volume conservation as well as the linearity of the mass attenuation coefficient with respect to the volume fraction (Eq. 1). The resulting system of equations consisting of a 3 × 3 system matrix (Eq. 2) represents a linear as well as convex problem and can thus be solved quickly and easily for the volume fractions $f_i$. Here $\mu _{i, 50/200}$ refer to the attenuation coefficient of the basis material *i* at an energy of 50keV and 200keV, respectively. And $\mu _{CT, 50/200}$ to the virtual monoE attenuation coefficients of the measurement. The condition of volume conservation is achieved here by the condition $f_1+f_2+f_3 = 1$^17^.

1 $$\begin{aligned} \mu _{CT,E}= & {} \sum _{i}f_i\cdot \mu _{i,E} \end{aligned}$$

2 $$\begin{aligned}{}&\begin{bmatrix} \mu _{1, 50} &{} \mu _{2, 50} &{}\mu _{3, 50}\\ \mu _{1, 200} &{} \mu _{2, 200} &{} \mu _{3, 200}\\ 1 &{} 1 &{} 1\\ \end{bmatrix} \left( \begin{array}{c} f_1\\ f_2\\ f_3\end{array}\right) = \left( \begin{array}{c} \mu _{CT,50}\\ \mu _{CT,200}\\ 1\end{array}\right) \end{aligned}$$

If there are more than three basic materials, this comparatively simple way can be modified by using the decomposition of human clots with histological analysis.

#### Material correction for histological analysis

Basically, this method achieves that two different imaging modalities can be correlated with each other mathematically. One modality is the DE-CT, which provides the virtual monoE images. The other modality is the histological analysis, which outputs the volume fraction of RBC, WBC and fibrin/platelets. Since the histological analysis overlooks materials such as iomeprol and formalin, the two results are usually different but still mathematically related. The simplest way to establish the relationship between these two modalities is to use a volume fraction representation. If the results of the DE-CT are interpreted as the sum of all actually occurring materials volume fractions $f_i^{CT}$, it must now apply that these can also be represented by the sum of all histological results $f_j^{Histo}$ normalized with $c_a$ and the basis materials $\tilde{f_k}$ not occurring in the histological analysis (Eq. 3). Here *i* represents all materials measured by the CT, *j* the materials that can only be measured visually by the histological analysis and *k* the missing materials that were not measurable by the histological analysis. Furthermore, due to volume conservation, for $n\in [0,1]$ it holds that $\sum _{i=1}^{4+n}f_i^{CT} = 1$ and $\sum _{j=1}^3 f_j^{Histo} = 1$. Thus, the correction factor $c_a$ can also be represented by Eq. (4). Substituting Eq. (2) into Eq. (4) and then changing from a volume fraction representation to an attenuation coefficient representation by multiplying each volume fraction value $f_i$ by the associated basis material attenuation coefficient $\vec {\mu }_i$ yields the transitions $\sum _{i=1}^{4+n}f_i^{CT} \longrightarrow \sum _{i=1}^{4+n}f_i^{CT}\cdot \vec {\mu }_i = \vec {\mu }^{CT}$ and $\sum _{j=1}^3 f_j^{Histo} \longrightarrow \sum _{j=1}^3 f_j^{Histo}\cdot \vec {\mu }_j = \vec {\mu }^{Histo}$ and $\sum _{k=4}^{4+n} \tilde{f_k}\longrightarrow \sum _{k=4}^{4+n} \tilde{f}_k\cdot \vec {\mu }_k$. Here $\vec {\mu }^{CT}$ denotes the attenuation vector at 50keV and 200keV determined using DE-CT. Whereas $\vec {\mu }^{Histo}$ denotes the attenuation vector that considers only the initial materials present in the histological findings. After presenting the equation in a more intuitive form and subsequent formation of the $l_2$ norm, a cost function (Eq. 5) is obtained, which is used to optimize to the volume fractions $\tilde{f}_k$.

3 $$\begin{aligned} \sum _{i=1}^{4+n}f_i^{CT}= & {} c_{a} \cdot \sum _{j=1}^3 f_j^{Histo} + \sum _{k=4}^{4+n} \tilde{f_k} \end{aligned}$$

4 $$\begin{aligned} c_a= & {} 1- \sum _{k=4}^{4+n} \tilde{f_k} \end{aligned}$$

5 $$\begin{aligned} \mathcal {L}_{\tilde{f_k}}= & {} \left| \left| \left( \vec {\mu }^{Histo}-\vec {\mu }^{CT}\right) - \sum _{k=4}^{4+n}\tilde{f}_k\left[ \vec {\mu }^{Histo} - \vec {\mu }_k\right] \right| \right| _{2}^{2} \end{aligned}$$

#### Optimization for basis material attenuation coefficient with fixated specimens

If only assumptions about the attenuation properties and the volume fractions of three of the basis materials are known, the attenuation coefficients of the three materials can be optimized. In addition, the method allows to consider at the same time another material like formalin with exactly known attenuation properties.

Thus, this method is divided into two sub steps. First, the value measured at the DE-CT must be adjusted for the fourth material. For this, similar to the method presented before, one can now subtract the volume fraction of the additional material $\tilde{f}_4$ from the CT value presented as the sum of all volume fractions and normalize this with $c_s$ (Eq. 6). This results in the volume fraction sum of the specimen composition without the fourth material. Since $\sum _{i=1}^4 f_{i}^{CT} = 1$ and $\sum _{j=1}^3 f_{j}^{'} = 1$ must hold here as well, one can express the normalization factor $c_s$ as Eq. (7). Moreover, an inverse relation holds between $c_s$ and $c_a$. Substituting Eq. (4) into Eq. (6) and moving from the volume fraction representation to the attenuation coefficient representation by $\sum _{i=1}^4 f_{i}^{CT} \longrightarrow \sum _{i=1}^4 f_{i}^{CT}\cdot \vec {\mu }_i = \vec {\mu }^{CT}$ and $\sum _{j=1}^3 f_{j}^{'} \longrightarrow \sum _{j=1}^3 f_{j}^{'}\cdot \vec {\mu }_j = \vec {\mu }^{'}$ are performed, then after a short transformation, an expression is obtained that relates measured DE-CT value to the attenuation coefficients adjusted for the fourth material (Eq. 8). The second part of the method refers to the correction of the basis materials. For this, the attenuation entries of the system matrix already listed in Eq. (2) are multiplied by individual correction factors $c_i$. This allows a correction for both density and mass attenuation. For each specimen *j* the difference between the corrected DE-CT value $\vec {\mu }_j^{'}$ and the calculated value from the system matrix and the histological result $\vec {f}_{j}^{Histo}$ can be calculated. $\vec {\mu }_j^{'}$ must be extended by one dimension with entry 1 to account for volume conservation. Summing now the $l_2$ normalized differentials of all specimens *j*, we obtain a cost function (Eq. 9) with which an optimization for the volume fraction $\tilde{f}_{4,j}$ and the correction factors $c_m$ with $m\in [1;6]$ can be obtained. Here it is important to emphasize again that the correction factors $c_m$ refer to the factors in Eq. (9). These allow an optimization of the ground truth absorption values of the base materials. Whereas $c_a$ as well as $c_s$ represent single factors, which allow the connection between DE-CT measurement and histological analysis.

6 $$\begin{aligned}&c_s\cdot \left( \sum _{i=1}^4 f_{i}^{CT} - \tilde{f}_4\right) = \sum _{j=1}^3 f_{j}^{'} \end{aligned}$$

7 $$\begin{aligned} c_s= & {} \frac{1}{1-\tilde{f}_4} = \frac{1}{c_a} \end{aligned}$$

8 $$\begin{aligned} \vec {\mu }_j^{'}= & {} \frac{\vec {\mu }_j^{CT} - \tilde{f}_{4,j}\cdot \vec {\mu }_4}{1 - \tilde{f}_{4,j}} \end{aligned}$$

9 $$\begin{aligned} \mathcal {L}_{\tilde{f}_{4,j}, c_m}= & {} \sum _j\left| \left| \left( \begin{array}{c} \vec {\mu }_j^{'} \\ 1 \end{array}\right) - \begin{bmatrix} c_1\cdot \mu _{1, 50} &{} c_2\cdot \mu _{2, 50} &{} c_3\cdot \mu _{3, 50}\\ c_4\cdot \mu _{1, 200} &{} c_5\cdot \mu _{2, 200} &{} c_6\cdot \mu _{3, 200}\\ 1 &{} 1 &{} 1\\ \end{bmatrix} \vec {f}_{j}^{Histo}\right| \right| _{2}^{2} \end{aligned}$$

The cost function (Eq. 9) poses a problem that is neither linear nor convex. Because of this, there can be multiple local minima, so a global optimizer must be used. For this study a simplicial homology global optimization (shgo)^36^ was used.

### Model test by iomeprol correction

#### Choice of chemicals

First, the model was verified on specimens of which the basis materials and the exact percentage composition are known. The first step is to find suitable basis materials.

The basic requirements for the selection of substances to be used as basis materials are that no precipitation reactions occur if all were mixed together and that they are readily soluble in water. At the same time, all substances should have the greatest possible difference in their attenuation properties and be easy to handle, since the specimens have to be transported.

Iomeprol, eosin Y disodiumsalt and sodium chloride in aqueous solution each fulfill these conditions very well. The iodine-containing iomeprol is not only the contrast agent used for contrast-enhanced CT in this study, but it is also a substance that is non-ionic. Therefore it cannot precipitate with other substances. The bromine-containing eosin Y disodiumsalt, on the other hand, is ionically bonded. However, since eosin Y disodiumsalt only precipitates with heavy anions such as phosphate or sulphate it fulfills the required conditions as well. Sodium chloride with chloride as the anion, is very suitable as a further substance since it fulfills all conditions and does not react or precipitates with the other substances. The main absorbing atoms iodine, bromine, chloride and the solvent water also ensures a different attenuation properties.

#### Specimen preparation

To prepare the basis solutions, 10.0 g of sodium chloride, 2.5 ml of an $400\, {\rm mg/ml}$ iomeprol solution and 2.0 g of eosin Y disodium salt were dissolved to the volume of 50 ml in distilled water in an 50 ml Erlenmeyer flask. Starting from the resulting basis solutions of $200 \, {\rm mg/ml}$ sodium chloride, $20 \, {\rm mg/ml}$ iomeprol and $40\, {\rm mg/ml}$ eosin Y disodiumsalt, five different concentrations were pipetted in each case (Table 5) into 1.5 ml sealable tubes. Using the measurement results from the DE-CT of these, linear regressions can be formed to show the accuracy of mixing but also to determine the values of the basis solutions more accurately.

Furthermore, several mixtures of a maximum of all three basis solutions as well as distilled water (Table 6) where pipetted into 1.5 ml closable tubes. Since the exact percent compositions are known, it can be pretended here that a modality corresponding to histological analysis in this case misses the iomeprol. The results of the DE-CT are then used to verify the iomeprol correction by using the model to try to optimize from the measured values and the volume composition (without iomeprol) to the true volume fractions of the solutions.

All tubes are then placed in two identical specimen holders that allow a measurement with the DE-CT of all specimen simultaneously. The composition of further measured samples and a representation of the sample holder can be found within the Supplementary Information as Tables S1, S2 and Fig. S1 online.

**Table 5:** Water-based diluted concentrations of the three basis materials (excluding distilled water itself) to determine the slope and intercept of a linear regression, which enables a more precisely calculation for the attenuation coefficients of the basis solutions.

Iomeprol$_{(aq.)}$ (mg/ml)	1.0	5.0	10.0	15.0	20.0
Eosin Y disodium salt$_{(aq.)}$ (mg/ml)	5.0	10.0	20.0	35.0	40.0
Sodium chloride$_{(aq.)}$ (mg/ml)	50.0	80.0	100.0	150.0	200.0

**Table 6:** Listing of all volumes pipetted into the 1.5ml tubes for each specimen.

Specimen	1	2	3	4	5	6
Eosin Y disodium salt$_{(aq.)}$ (ml)	0.5	0.3	0.1	0.05	0.1	0.005
Iomeprol$_{(aq.)}$ (ml)	0.0	0.3	0.0	0.05	0.1	0.003
Water (pure) (ml)	0.5	0.3	1.0	1.0	0.5	0.5
Sodium chloride$_{(aq.)}$ (ml)	0.5	0.3	0.0	0.4	0.5	0.5

### Iomeprol correction for human clots

#### Extraction and preparation of the clots

72 patients with acute ischemic stroke, and one patient with a sinus vein thrombosis were treated by mechanical thrombectomy. Clot material could then be extracted and immediately placed in tubes containing neutral buffered 3.5–3.7$\%$ formalin solution for fixation and storage. Except for one patients, which had two acute strokes independently of each other and thus two clots were extracted, all 73 acute stroke clots (12 for optimization and validation and 61 for testing of the material decomposition approach) were from different patients each (median of age: 79, interquartile range (IQR) (Q1 and Q2): 68–85, female fraction: 58.9$\%$). Afterwords CT scans of the clots were performed. Therefore a special specimen holder, in which two tubes or two specimens can be placed simultaneously, is then put on the patient desk with the aid of a cardboard tray so that the specimen holder does not move and at the same time an inclined plane is formed. Thus, the specimens remain constantly at the bottom of the tubes and do not move during the measurement (Fig.1). Further images of the sample holder can be found within the Supplementary Information as Figs. S5 and S6.

#### Histological analysis

For the application of the method on human clots, it is obvious to perform the established histological analysis to obtain the composition of RBC, WBC and fibrin/platelets, which was performed for each of the 73 specimens (12 for optimization and validation and 61 for testing of the material decomposition approach). This involves cutting the clots into many approximately $1$ to $2 \,\upmu m$ thick sections, which are then routinely stained with hematoxylin–eosin (HE) and Elastica van Gieson. Afterwards the slides were scanned at high resolution by a light microscope and digitally stored^37^. Due to the cutting of the samples, it is important that the histological analysis was performed after the DE-CT measurement. Since afterwards, the samples can no longer be measured by CT, as they are then destroyed. For the evaluation of the percentage distribution the software Orbit Image Analysis (Version 3.64, Idorsia Pharmaceuticals Ltd., Allschwil, Schwitzerland) was used. This free open source software achieves the segmentation by a machine learning algorithm. Sometimes cracks or overlaps occur in the analyzed histosections, which would affect the result. These were manually left out for the analysis to prevent high errors. Since fibrin and platelets cannot be visually distinguished in HE staining, both are considered as one conglomerate for this study^18^ A graphical representation of the analysis based on one sample section can be found within the Supplementary Information as Fig. S7 online.

#### Basis materials optimization

This optimization refers to the formulas (6)–(9). Here *j* represents the sample number and *i* represents the abbreviations RBC, WBC, fibrin/platelets, and formalin. In order to significantly limit the optimization environment and thus also reduce the duration of the optimization of the correction factors $c_m$ and the volume fraction of formalin corresponding to $\tilde{f}_{4,j}$ in Eq. (9), it is important to predetermine the attenuation properties as precisely as possible.

For the hemoglobin that is within the RBC, the sum of all elements occurring in the two $\alpha$-globine, two $\beta$-globine (see Supplementary Information Fig. S4, Tables S3 and S4 online) and four hem-groups which are all part of the quarterly structure is summed up to a total chemical sum formula^38,39^. The density of the hemoglobin is extracted from the mean corpuscular hemoglobin concentration (MCHC) of each patient individually. This value is routinely determined in patients when blood count is performed. For fibrin the chemical sum formula and the density can be looked up at PubChem^24^. It should be noted here that only the properties of fibrin were looked up or determined. As known from histological analysis, fibrin and platelets are seen as one material due to HE staining. Thus, the platelets are not considered for chemical sum formula assumption. However, the algorithm allows to optimize from fibrin to fibrin/platelets later on. The WBC are substituted by soft tissue assuming that the difference in X-ray attenuation between the is negligible. The chemical sum-formula such as the density of formalin the specimen are fixated can also be looked up. With the knowledge of the chemical sum formula and the density of the RBC, WBC, fibrin and formalin the attenuation coefficients at 50keV and 200keV are calculated or directly looked up by using the United States National Institute of Technology and Standards (NIST) X-ray Mass Attenuation Coefficients Database^23^.

For the basis material optimization of RBC, WBC and fibrin/platelets 12 of all 73 specimen where used which do not have iomeprol diffused to reduce the dimensioning of the model. To reduce this further and make the algorithm faster, the 12 specimen are grouped in three groups containing four specimen each. The correction factors for WBC were set equal to 1, since WBC do not contribute much to the attenuation and also the dimensioning of the problem had to be further reduced. Each group is then optimized separately with the in-house developed algorithm resulting in three different results for $c_m$ and the volume fraction of formalin. The end result is then the weighted mean of these. Whereas, the weighting coefficients are the inverse of the value of the cost-function at the minimum.

By using the final results of the $c_m$ these can now be multiplied with the assumptions of the attenuation resulting in the final optimized attenuation coefficient of RBC, WBC and fibrin/platelets that are used for the following iodine correction of the other specimen not used for this basis material optimization.

#### Iodine correction

This optimization refers to the Eqs. (3), (4) and (5). Here *i* corresponds to the abbreviations of RBC, WBC, fibrin/platelets, formalin and iomeprol. *j* corresponds to RBC, WBC and fibrin/platelets only and *k* corresponds to formalin and iomeprol. The final goal is now to use the knowledge of the basis material attenuation properties to do an iodine as well as an formalin correction for the 61 remaining specimens.

The iomeprol attenuation can be calculated by using the NIST database and looking up the chemical sum formula and density^23^. Then, the attenuation properties of all basis materials are known and the correction for the iomeprol can be applied using the cost-function (Eq. 5) in consideration of formalin as fifth material.

### CT image acquisition

73 human clots and six test mixtures consisting of water-based solutions of eosin Y disodium salt, sodium chloride, iomeprol and pure water were measured with the DL-CT (IQon Spectral CT, Philips Healthcare, Best, The Netherlands). The test mixtures were fixated and positioned using a suitable holder. This allows all test specimens to be measured simultaneously. The clots were measured using a holder suitable for these specimens (Fig.1b). This holder allows two clots specimens to be measured simultaneously. All specimens were placed as centrally as possible with respect to the gantry for measurement. Each scan was performed with a tube voltage of 120 kVp and an exposure of 500 mAs. The results of the front and rear detector layer were then used by the manufacturer’s internal software (IQon—Spectral CT powered by iPatient v4.7.5.43531) to calculate spectral information, which is stored as a spectral base images (SBI). The CT dose index (CTDI$_{vol}$) per scan calculated by the scanner was 85.9mGy for every specimen and resulted in a mean dose length product (DLP) of 215 mGy cm for the clots and 4766.5mGy cm for the test specimens. The Image matrix of 512 × 512 pixel, pitch of 0.328 s, rotation time of 0.75 s and a detector configuration of 64 × 0.625 mm was used for all specimens.

Reconstruction of SBI using projection data from the front and rear detector slices, a Brain Sharp (UC) filter, spectral plane 2, and an axial slice thickness of 0.8 mm was performed using Philips internal software (IQon—Spectral CT powered by iPatient v4.7.5.43531). The MonoE images at 50 keV and 200 keV, important for this study, were mathematically calculated using the SBI and the manufacturer-specific software (Intelli Space Portal v.9.0.1, Philips Healthcare, The Netherlands). Due to the mathematical determination of these images, they are so called virtual monoE images created in Hounsfield Units (HU).

### Postprocessing

After the CT image acquisition, the data was loaded into a desktop software (3D slicer image computing platform)^40^. The region of interest (ROI) was then segmented using the available tools and the mean attenuation for the virtual monoE images was generated for the voxels considered. In addition, the clinically relevant HU value was normalized to the physical relevant value with Eq. (10).

10 $$\begin{aligned} \mu (E) = \frac{HU(E)\cdot \mu _{water}(E)}{1000[HU]}+\mu _{water}(E) \end{aligned}$$

### Implementation

The algorithm was implemented on python and executed with Ipython (version 3.7)^41^. For the visual representation the package matplotlib.pyplot was used and for general mathematical implementation the package NumPy^42,43^.

### Ethical approval

This retrospective study was approved by the Ethics Committee of the Technical University of Munich. Due to the retrospective nature of the study, the Ethics Committee assumes that all data and thrombi of the patients to be included are already available. Therefore written informed consent was waived by the Ethics Committee of the Technical University of Munich for this retrospective analysis. All experiments were performed in accordance with the relevant guidelines and regulations.

## Supplementary Information

Supplementary Information.

Supplementary Table S6.

Supplementary Table S7.

## Data Availability

The datasets generated and/or analysed during the current study are not publicly available according to the regulations on data protection and in accordance with the ethical standards but are available from the corresponding author on reasonable request.
