# Supplementary material for: Five material tissue decomposition by dual energy computed tomography
Source: Sci Rep. 2022 Oct 12;12:17117. doi: 10.1038/s41598-022-21193-5 (PMC9556609; doi:10.1038/s41598-022-21193-5)
Supplement: Supplementary file 1 — Supplementary Information. [file 41598_2022_21193_MOESM1_ESM.pdf]

Supplementary file for:

Five Material Tissue Decomposition by Dual Energy Computed Tomography

Maximilian E. Lochschmidt[1,*,+], Melina Gassenhuber[2,+], Isabelle Riederer[5], Johannes Hammel[1,2], Lorenz Birnbacher[1,2], Madleen Busse[1,3], Tobias Boeckh-Behrens[5], Benno Ikenberg[6], Silke Wunderlich[6], Friederike Liesche-Starnecker[7], Jürgen Schlegel[7], Marcus R. Makowski[2], Claus Zimmer[5], Franz Pfeiffer[1,2,3,4,+], and Daniela Pfeiffer[2,4,+]

[1]Chair of Biomedical Physics, Department of Physics, School of Natural Sciences, Technical University of Munich, 85748 Garching, Germany
[2]Department of Diagnostics and Interventional Radiology, School of Medicine, Klinikum rechts der Isar, Technical University of Munich, 81675 München, Germany
[3]Munich Institute of Biomedical Engineering, Technical University of Munich, 85748 Garching, Germany
[4]Institute for Advanced Study, Technical University of Munich, 85748 Garching, Germany
[5]Department of Diagnostic and Interventional Neuroradiology, School of Medicine, Klinikum rechts der Isar, Technical University of Munich, 81675 München, Germany
[6]Department of Neurology, School of Medicine, Klinikum rechts der Isar, Technical University of Munich, 81675 München, Germany
[7]Department of Neuropathology, School of Medicine, Klinikum rechts der Isar, Technical University of Munich, 81675 München, Germany
[*]maximilian.lochschmidt@tum.de
[+]These authors contributed equally to this work

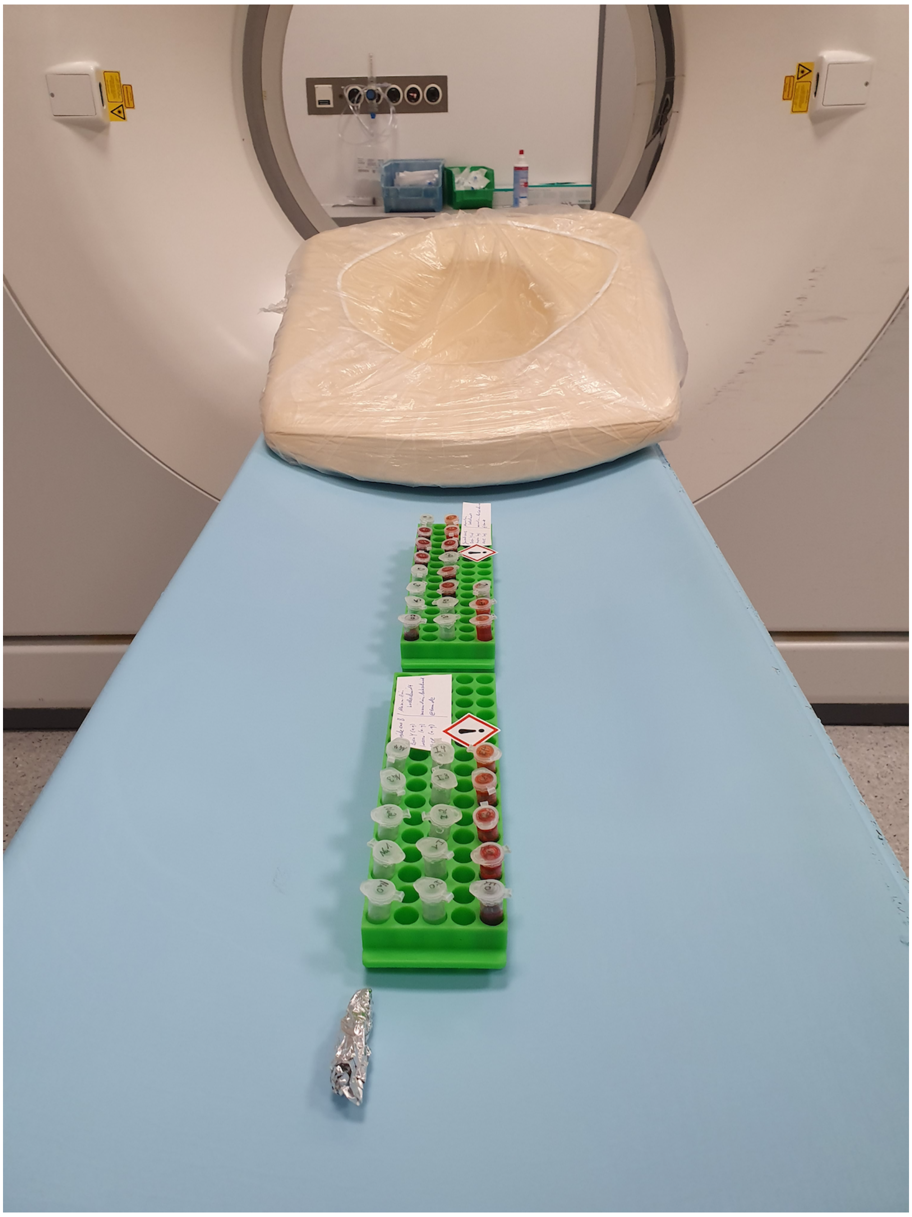

Figure S 1. Test samples placed into sample holders before the measurement.

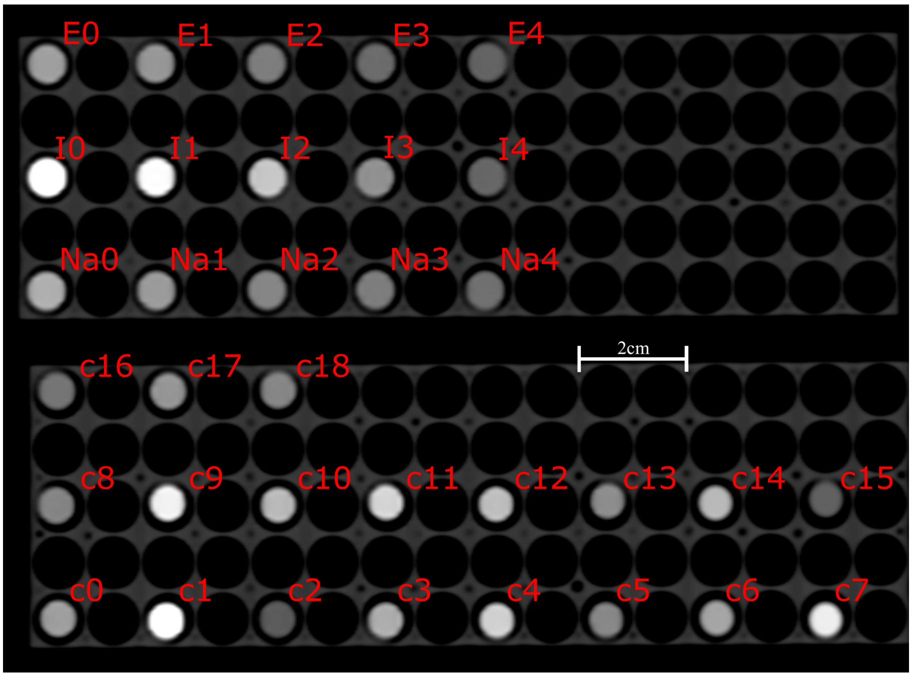

Figure S 2. View of all measured test samples at a virtual monoenergetic energy of 50keV. Here E stands for the eosin, I for the iomeprol and Na for the sodium chloride mixture. Samples abbreviated with c represent mixtures of all these three base material mixtures as well as pure water. The number after each material abbreviation is used to assign the samples.

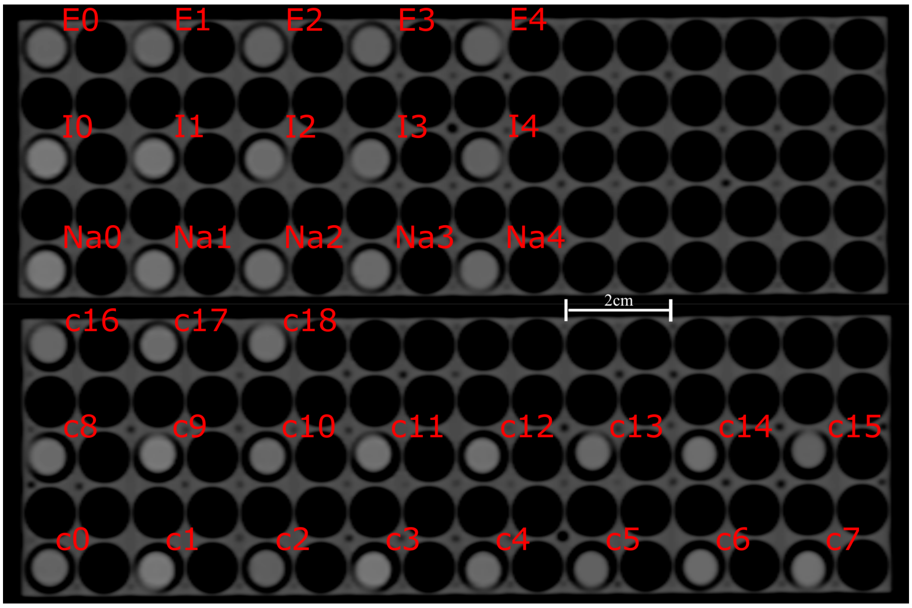

Figure S 3. View of all measured test samples at a virtual monoenergetic energy of 200keV. Here E stands for the eosin, I for the iomeprol and Na for the sodium chloride mixture. Samples abbreviated with c represent mixtures of all these three base material mixtures as well as pure water. The number after each material abbreviation is used to assign the samples.

Mixtures	c0	c1	c2	c3	c4	c5	c6	c7	c8	c9	c10	c11	c12	c13	c14	c15	c16	c17	c18
Eosin Y disodiumsalt [ml]	1.0	0.0	0.0	0.0	1.0	1.0	1.0	0.0	0.0	0.0	0.5	0.5	0.0	0.5	0.3	0.1	0.05	0.1	0.005
Iomeprol [ml]	0.0	1.0	0.0	0.0	0.5	0.0	0.0	1.0	0.0	0.5	0.5	0.5	0.5	0.0	0.3	0.0	0.05	0.1	0.003
Water [ml]	0.0	0.0	1.0	0.0	0.0	0.5	0.0	0.5	0.5	0.0	0.5	0.0	0.5	0.5	0.3	1.0	1.0	0.5	0.5
Sodium chloride [ml]	0.0	0.0	0.0	1.0	0.0	0.0	0.5	0.0	0.5	0.5	0.0	0.5	0.5	0.5	0.3	0.0	0.4	0.5	0.5

Table S 1. Absolute volumes for the samples c_i, which are intended for testing the theory. The sample indices c13, c14, c15, c16, c17 and c18 correspond to the indexing 1, 2, 3, 4, 5 and 6 in the manuscript. The setup of the measurement and the monoenergetic CT images of the results are shown in Figure S1, Figure S2 and Figure S3.

Mixtures	c0	c1	c2	c3	c4	c5	c6	c7	c8	c9	c10	c11	c12	c13	c14	c15	c16	c17	c18
Eosin Y disodiumsalt	1.000<br>(1.000)	0.000<br>(0.000)	0.000<br>(0.000)	0.000<br>(0.000)	$0.\overline{6}$<br>(1.000)	$0.\overline{6}$<br>$(0.\overline{6})$	$0.\overline{6}$<br>$(0.\overline{6})$	0.000<br>(0.000)	0.000<br>(0.000)	0.000<br>(0.000)	$0.\overline{3}$<br>(0.500)	$0.\overline{3}$<br>(0.500)	0.000<br>(0.000)	$0.\overline{3}$<br>$(0.\overline{3})$	0.250<br>$(0.\overline{3})$	$0.\overline{09}$<br>$(0.\overline{09})$	$0.0\overline{3}$<br>$(\frac{1}{29})$	$0.08\overline{3}$<br>$(0.\overline{09})$	$\frac{5}{1008}$<br>$(\frac{1}{201})$
Iomeprol	0.0	1.0	0.0	0.0	$0.\overline{3}$	0.0	0.0	$0.\overline{6}$	0.0	0.5	$0.\overline{3}$	$0.\overline{3}$	$0.\overline{3}$	0.0	0.250	0.0	$0.0\overline{3}$	$0.08\overline{3}$	$\frac{1}{336}$
Water	0.0<br>(0.0)	0.0<br>(0.0)	1.0<br>(1.0)	0.0<br>(0.0)	0.0<br>(0.0)	$0.\overline{3}$<br>$(0.\overline{3})$	0.0<br>(0.0)	$0.\overline{3}$<br>(1.0)	0.5<br>(0.5)	0.0<br>(0.0)	$0.\overline{3}$<br>(0.5)	0.0<br>(0.0)	$0.\overline{3}$<br>(0.5)	$0.\overline{3}$<br>$(0.\overline{3})$	0.25<br>$(0.\overline{3})$	$0.\overline{90}$<br>$(0.\overline{90})$	$0.\overline{6}$<br>$(\frac{20}{29})$	$0.41\overline{6}$<br>$(0.\overline{45})$	$\frac{125}{252}$<br>$(\frac{100}{201})$
Sodium chloride	0.0<br>(0.0)	0.0<br>(0.0)	0.0<br>(0.0)	1.0<br>(1.0)	0.0<br>(0.0)	0.0<br>(0.0)	$0.\overline{3}$<br>$(0.\overline{3})$	0.0<br>(0.0)	0.5<br>(0.5)	0.5<br>(1.0)	0.0<br>(0.0)	$0.\overline{3}$<br>(0.5)	$0.\overline{3}$<br>(0.5)	$0.\overline{3}$<br>$(0.\overline{3})$	0.25<br>$(0.\overline{3})$	0.0<br>(0.0)	$0.2\overline{6}$<br>$(\frac{8}{29})$	$0.41\overline{6}$<br>$(0.\overline{45})$	$\frac{125}{252}$<br>$(\frac{100}{201})$

Table S 2. Representation of the true volume fractions and the simulated fractions simulating if iomeprol would not be taken into account. The sample indices c13, c14, c15, c16, c17 and c18 correspond to the indexing 1, 2, 3, 4, 5 and 6 in the manuscript. The setup of the measurement and the monoenergetic CT images of the results are shown in Figure S1, Figure S2 and Figure S3.

```
>3NL7_1|Chain A|Hemoglobin subunit alpha|Homo sapiens (9606)
VLSPADKTNVKAAWGKVGAHAGEYGAEALERMFLSFPTTKTYFPHFDLSHGSAQVKGHGKKVADALTNAVAHVDDMPNALSALSDLHAHKLRVDPVNFKLLSHCLLVTLAAHLPAEFTPAVHASLDKFLASVSTVLTSKYR
>3NL7_2|Chain B|Hemoglobin subunit beta|Homo sapiens (9606)
VHLTPEEKSAVTALWGKVNVDEVGGEALGRLLVVYPWTQRFFESFGDLSTPDAVMGNPKVKAWGKKVLGAFSDGLAHLDNLKGTFATLSELHCDKLHVDPENFRLLGNVLVCVLAHHFGKEFTPPVQAAYQKVVAGVANALAHKYH
```

Figure S 4. Exact amino acids sequence of the subunits α-globuline (upper chain) and β-globuline (lower chain) that form the quaternary structure of human hemoglobine[1]. The abbreviations and the atomic content of each amino acid can be looked up in Table S3 and Table S4.

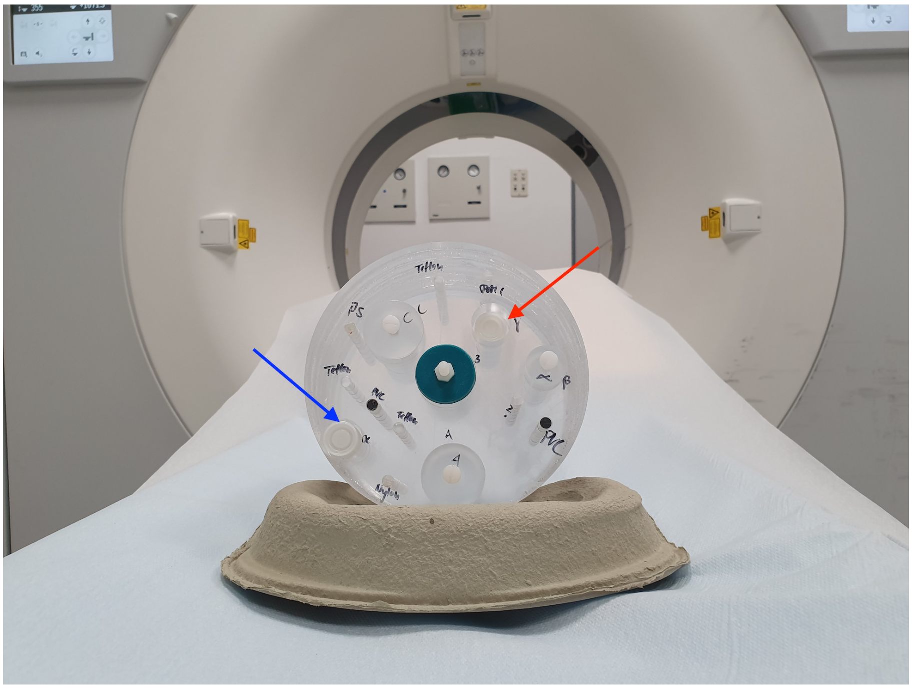

Figure S 5. Illustration of the sample holder for the tubes with formalin fixated human clots. The two holes into which the sample tubes can be inserted are marked by the blue and red arrows.

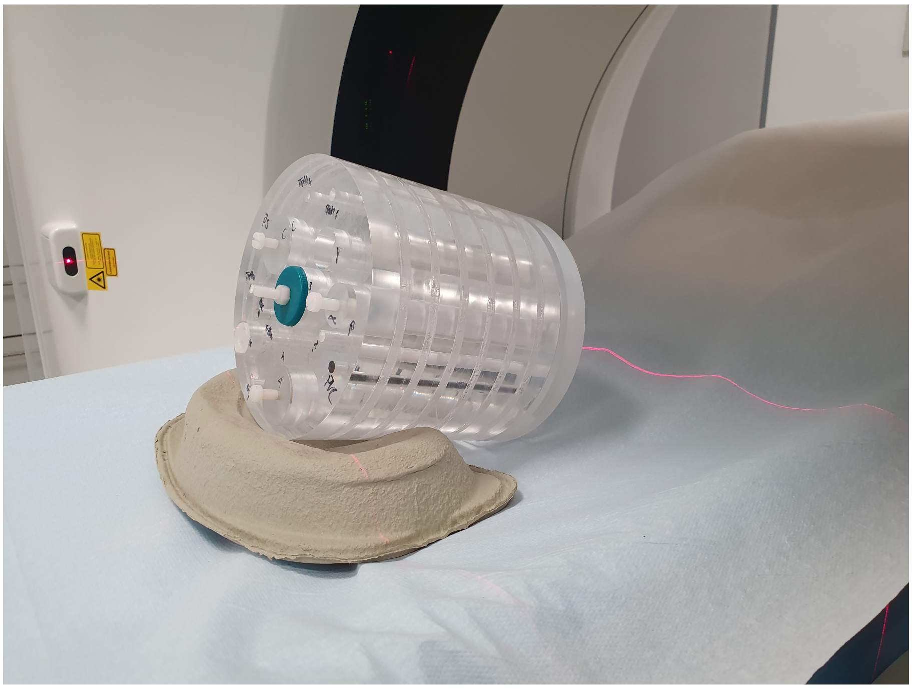

Figure S 6. Side view of the sample holder (Figure S5) to illustrate the slightly inclined plane that ensures that clots remain at the bottom of the tubes during measurement.

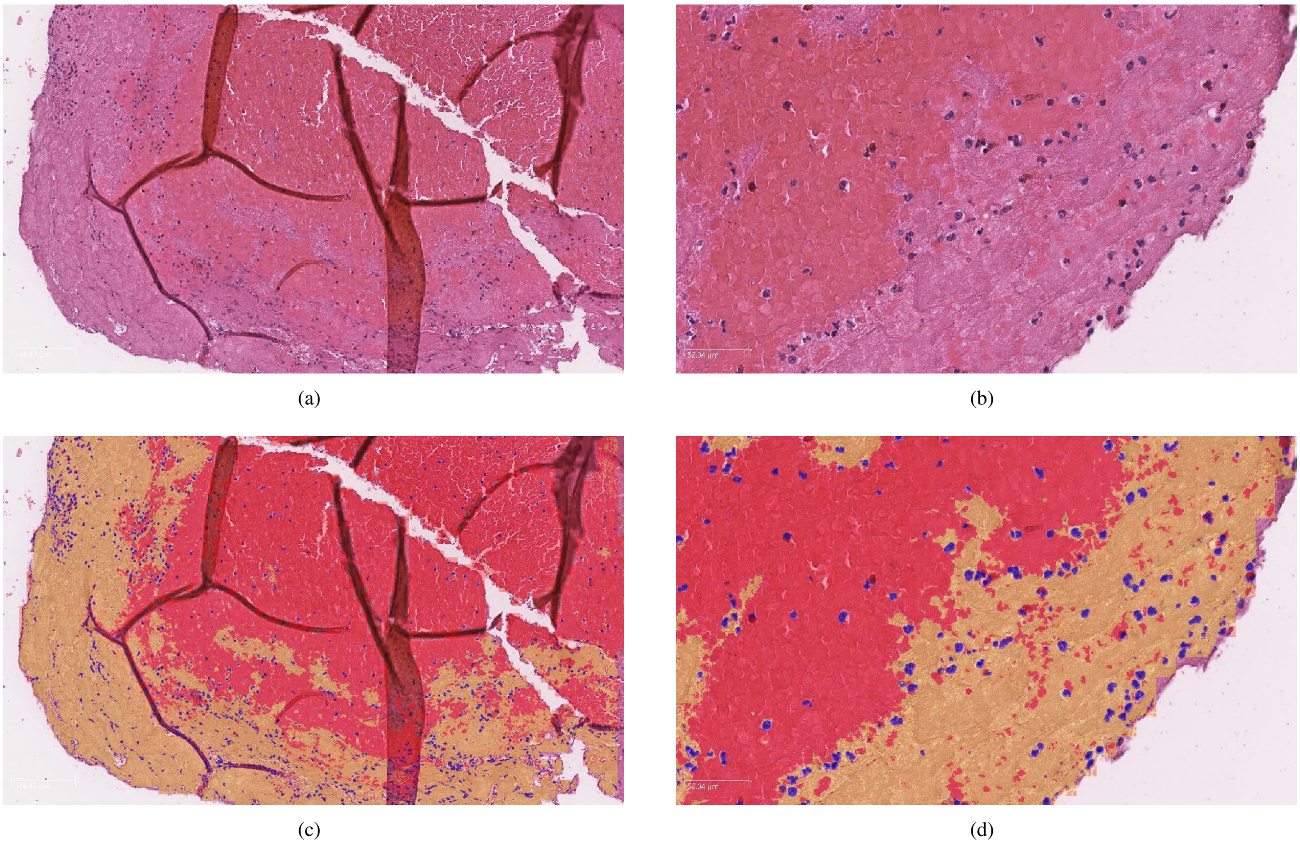

(a) (b) (c) (d)

Figure S 7. **(a,b)** Example of a histological section after HE- as well as Elastica van Gieson-staining as well as **(c,d)** the same section after segmentation of WBC (blue segment), RBC (red segment) and Fibrin/Platelets (yellow segment) by the program.

Name	Abbreviation	C	H	N	O	S
Alanine	A	3	7	1	2	0
Arginine	R	6	14	4	2	0
Asparagine	N	4	8	2	3	0
Aspartic acid	D	4	7	1	4	0
Cysteine	C	3	7	1	2	1
Glutamine	Q	5	10	2	3	0
Glutamic acid	E	5	9	1	4	0
Glycine	G	2	5	1	2	0
Histidine	H	6	9	3	2	0
Isoleucine	I	6	13	1	2	0
Leucine	L	6	13	1	2	0
Lysine	K	6	14	2	2	0
Methionine	M	5	11	1	2	1
Phenylalanine	F	9	11	1	2	0
Proline	P	5	9	1	2	0
Serine	S	3	7	1	3	0
Threonine	T	4	9	1	3	0
Tryptophan	W	11	12	2	2	0
Tyrosine	Y	9	11	1	3	0
Valine	V	5	11	1	2	0

Table S 3. Relevant amino acids, their typical abbreviations and their carbon C, hydrogen H, nitrogen N, oxigen O and sulfur S content. Data taken from[2].

Amino acid	α-globuline	β-globuline
A	21	15
R	3	3
N	4	6
D	8	7
C	1	2
Q	1	3
E	4	8
G	7	13
H	10	8
I	0	0
L	18	18
K	11	11
M	2	1
F	7	8
P	7	7
S	11	5
T	9	7
W	1	3
Y	3	3
V	13	18

Table S 4. Amino acid composition of the two protein structures α- and β-globuloins found in hemoglobin. The carbon C, hydrogen H, nitrogen N, oxygen O and sulfur S content as well as the corresponding full names of the abbreviated amino acids can be looked up in Table S3. Data taken from[1].

Basis Name	Atomic Sum Formula or Assumption	$\left(\frac{\mu}{\rho_m}\right)_{50\text{keV}}$	$\left(\frac{\mu}{\rho_m}\right)_{200\text{keV}}$	$\rho_m[\frac{g}{ml}]$
RBC	$C_{2964}H_{5654}Fe_4N_{778}O_{1376}S_{12}$	0.2150	0.1325	MCHC (0.30-0.39)
WBC	Soft-Tissue	0.2230	0.1360	1.00
Fibrin	$C_5H_{11}N_3O_2$	0.2074	0.1324	1.395
Iomeprol	$C_{17}H_{22}I_3N_3O_8$	6.1419	0.2457	2.281
Formalin	CH_2O	0.2110	0.1314	0.8153

Table S 5. Chemical sum formula or an assumption of the composition for the basis materials RBC, WBC, fibrin, iomeprol and formalin. The mass attenuation values $\left(\frac{\mu}{\rho_m}\right)_E$ for the energies 50keV and 200keV calculated using the NIST database are listed here. In addition, the assumed base material densities are given[3]. It should be noted here that the MCHC value represents the mean hemoglobin concentration in the RBCs, which is routinely determined by blood count. The long sum formula for the RBCs has been determined based on Table S3, Table S4, Figure S4 and using PubChem[4].

References

1. Berman, H. M. *et al.* The Protein Data Bank, DOI: https://doi.org/10.1093/nar/28.1.235 (2000).
2. Dixon, H. B. F. Nomenclature and Symbolism for Amino Acids and Peptides: Recommendations 1983. *Eur. J. Biochem.* **138**, 9–37, DOI: https://doi.org/10.1111/j.1432-1033.1984.tb07877.x (1984).
3. Hubbell, J. H. & Seltzer, S. M. Tables of X-Ray Mass Attenuation Coefficients and Mass Energy-Absorption Coefficients (version 1.4), DOI: https://dx.doi.org/10.18434/T4D01F (2004).
4. Kim, S. *et al.* PubChem in 2021: new data content and improved web interfaces. *Nucleic Acids Res.* **49**, D1388–D1395, DOI: 10.1093/nar/gkaa971 (2020). https://academic.oup.com/nar/article-pdf/49/D1/D1388/35363961/gkaa971.pdf.
